# Identification and Expression Profiling of the Cytokinin Synthesis Gene Family *IPT* in Maize

**DOI:** 10.3390/genes16040415

**Published:** 2025-03-31

**Authors:** Congcong Chen, Yujie Yan, Dongxiao Li, Weixin Dong, Yuechen Zhang, Peijun Tao

**Affiliations:** 1 College of Agriculture, Hebei Agricultural University, Baoding 071001, China; 15733227268@163.com (C.C.); yyj1853267@163.com (Y.Y.); lidongxiao.xiao@163.com (D.L.); 2 Teaching Support Department, Hebei Open University, Shijiazhuang 050080, China; dongweixin.yuxin@163.com

**Keywords:** maize, *IPT* gene family, cytokinin, abiotic stress, expression pattern

## Abstract

Isopentyltransferase (*IPT*) is a key rate-limiting enzyme in cytokinin synthesis, playing a crucial role in plant growth, development, and response to adverse conditions. Although the IPT gene family has been studied in various plants, comprehensive identification and functional characterization of *IPT* genes in maize (*Zea mays*) remain underexplored. In this study, ten *IPT* gene family members (*ZmIPT1*–*ZmIPT10*) were identified in the maize genome, and their gene structure, physicochemical properties, evolutionary relationships, expression patterns, and stress response characteristics were systematically analyzed. The *ZmIPT* genes were found to be unevenly distributed across six chromosomes, with most proteins predicted to be basic and localized primarily in chloroplasts. Phylogenetic analysis grouped the *ZmIPT* family into four subfamilies, showing close evolutionary relationships with rice IPT genes. Conserved motif and gene structure analyses indicated that the family members were structurally conserved, with five collinear gene pairs being identified. Ka/Ks analysis revealed that these gene pairs underwent strong purifying selection during evolution.Cis-element analysis of promoter regions suggested that *ZmIPT* genes are widely involved in hormone signaling and abiotic stress responses. Tissue-specific expression profiling showed that *ZmIPT5*, *ZmIPT7*, and *ZmIPT8* were highly expressed in roots, with *ZmIPT5* exhibiting consistently high expression under multiple abiotic stresses. qRT-PCR validation confirmed that *ZmIPT5* expression peaked at 24 h after stress treatment, indicating its key role in long-term stress adaptation. Protein interaction analysis further revealed potential interactions between *ZmIPT5* and cytokinin oxidases (*CKX1*, *CKX5*), as well as FPP/GGPP synthase family proteins, suggesting a regulatory role in cytokinin homeostasis and stress adaptation. Overall, this study provides comprehensive insights into the structure and function of the *ZmIPT* gene family and identifies *ZmIPT5* as a promising candidate for improving stress tolerance in maize through molecular breeding.

## 1. Introduction

Cytokinins are essential for numerous physiological processes in plants, including apical dominance, branching, tillering, leaf senescence, photosynthesis, and stress responses [1,2,3]. Among the key enzymes involved in cytokinin biosynthesis, isopentenyltransferase (*IPT*) serves as a rate-limiting enzyme, playing a central role in regulating these processes [4,5]. IPT catalyzes the transfer of the isopentenyl group from dimethylallyl diphosphate (DMAPP) to adenosine monophosphate, ultimately leading to cytokinin synthesis, which modulates plant growth and development. *IPT* can be classified into two main types: ATP/ADP IPT, which modifies adenine in tRNA; and tRNA-IPT, which specifically transfers the isopentenyl group to adenosine monophosphate. This classification highlights the diversity of *IPT* genes across different plant cells and suggests potential functional differences [6,7,8].

Numerous studies have demonstrated the crucial role of *IPT* in plant stress responses [9,10,11]. Under heat stress conditions, cytokinin accumulation induced by *IPT* genes enhances plant thermotolerance by regulating stomatal opening and the expression of photosynthesis-related proteins and transcripts. In transgenic sugarcane, the overexpression of *IPT* has been shown to increase cold tolerance [12], with similar findings reported in transgenic eggplants [13]. Furthermore, in rice, transgenic *IPT* plants exhibited significantly higher cytokinin and chlorophyll content under drought stress, thereby improving their tolerance to osmotic stress and drought. This phenomenon has also been documented in transgenic Arabidopsis [14], tobacco [15], and chickpea [16]. Additionally, overexpression of *IPT* under the control of a stress-inducible promoter (pSARK::IPT) has been linked to delayed drought-induced senescence and enhanced drought resistance in crops such as peanut and cotton [17,18,19,20]. Transgenic plants expressing SAG12-IPT under phosphate starvation conditions demonstrated increased acidic nitrogen and phosphatase activities, indicating the pivotal role of *IPT* in enhancing plant adaptation to nitrogen- and phosphorus-limited environments [21,22]. IPT genes also significantly contribute to enhancing plant resistance to herbivory by promoting cytokinin synthesis in plant cells. Studies indicate that *IPT* gene promoters, in conjunction with protease inhibitor II genes, can suppress the growth and development of aphids on peach trees [23]. These findings underscore the essential function of *IPT* genes in mitigating stress-related damage, thereby improving plant resilience in challenging environments.

Maize (*Zea mays*) is a crucial crop for global food security, economic stability, and animal feed production. As the global population continues to rise, the demand for food increases, making the improvement of maize yield a critical strategy for ensuring food security [24]. However, climate change and environmental stressors, particularly extreme temperatures, drought, and water scarcity, significantly impact maize growth and development, severely limiting its yield potential. Recent research has underscored the essential role of cytokinins in maize’s response to environmental stresses [25,26]. Cytokinins not only enhance maize’s adaptability to drought and salinity stress by regulating root growth, leaf development, and water use efficiency, but they also play a significant role in modulating antioxidant enzyme systems and improving stress resistance [27,28,29]. Therefore, a comprehensive understanding of the functions of cytokinins in maize’s stress resistance mechanisms is of substantial theoretical and practical significance for enhancing maize resilience and advancing global food production.

To date, several plant species have been identified to contain *IPT* genes, including Arabidopsis [30], rice [31], apple [32], tomato [33], and soybean [34]. However, a comprehensive examination of the *IPT* gene family in maize remains lacking. In this study, we employed bioinformatics techniques to identify the members of the maize *IPT* family and conducted a detailed analysis of their gene structures, evolutionary relationships, conserved motifs, syntenic relationships, cis-acting elements, and expression patterns. This research provides a solid theoretical foundation for further investigations into the functional roles of *IPT* family members in maize.

## 2. Materials and Methods

### 2.1. Genome-Wide Identification and Physicochemical Properties Analysis of the Maize IPT Gene Family Members

First, we retrieved the IPT protein domain file in the Hidden Markov model format from the Pfam database (http://pfam.xfam.org/ accessed on 1 January 2020). We then used HMMER 3.0 to generate the maize IPT protein sequence file. Next, we visited the NCBI database (https://www.ncbi.nlm.nih.gov/, accessed on 1 September 2024) to further identify the protein domain linked to the candidate gene. After this process, the amino acid sequence devoid of the IPT domain was ultimately acquired. The amino acid length, molecular weight, isoelectric point, and other relevant indicators of the candidate IPT family members were analyzed using ProtParam and ProtScale in ExPASy (https://web.expasy.org/protparam/, accessed on 1 September 2024). Subcellular localization was assessed through the WoLF PSORT Prediction tool (https://wolfpsort.hgc.jp/, accessed 1 September 2024), while the gene’s localization data was visualized with TBtools-II software to identify the chromosome’s length and position that corresponds to the maize *IPT* gene information. We downloaded the IPT protein sequences of Arabidopsis, rice, and soybean from the EnsemblPlants database (https://plants.ensembl.org/index.html, accessed on 1 September 2024). We utilized MEGA7 for multi-sequence alignment and employed iTOL for the beautification of the phylogenetic tree. Subsequently, we extracted the sequence 2000 bp upstream of the transcription start site of the candidate IPT family gene in maize using TBtools software. The Plant CARE tool (http://bioinformatics.psb.ugent.be/webtools/plantcare/html/, accessed on 1 September 2024) will be utilized to anticipate the cis-acting elements within the promoter region for a member of the maize *IPT* gene family using this 2000 bp sequence. Additionally, we performed collinear visual analysis of the *IPT* gene within the maize genome using TBtools software. Finally, we utilized PPRD (http://ipf.sustech.edu.cn/pub/plantrna, accessed 1 September 2024) to investigate and gather data on the expression levels of ZmIPTs within diverse maize tissues and in response to different abiotic stress conditions.

### 2.2. Plant Material and Stress Treatments

The study took place in June 2024 at Hebei Agricultural University’s Agricultural College, located in Baoding, Hebei (38.82 °N, 115.45 °E). The maize variety Zhengdan 958, provided by Pioneer Seed Industry, was used as the experimental material. The seedling matrix consisted of a mixture of vermiculite and nutrient soil in a volume ratio of 3:1, and the seedlings were cultivated in an incubator set to 25 °C, with a relative humidity of 65% and a photoperiod of 16 h light and 8 h dark. When the maize seedlings reached the two-leaf and one-center stage, those exhibiting consistent growth potential were selected for follow-up experiments.

Tissue expression analysis was conducted on the root, shoot, and leaf parts of maize seedlings to assess gene expression.

Five treatment conditions were established for the abiotic stress experiments: (1) CK, which involved maize seedlings under standard management practices; (2) Heat stress, where the incubator temperature was maintained at 42 °C during the day and 25 °C at night; (3) Cold stress, with the temperature in the incubator adjusted to 4 °C during the day and 10 °C at night; (4) Drought stress, achieved by employing a 20% PEG-6000 solution; (5) Salt stress, where the roots were irrigated with a 200 mM NaCl solution. For each treatment and control group, three biological replicates were performed. Samples were taken at 0, 1, 6, and 12 h, with blade samples rapidly frozen in liquid nitrogen and preserved in a −80 °C freezer for later analysis.

### 2.3. Total RNA Extraction, cDNA Reverse Transcription, and Quantitative Real-Time PCR Analysis

RNA extraction was conducted using a kit from Beijing Huayueyang Co., Ltd. (Beijing, China). Reverse transcription and real-time fluorescence quantitative PCR analyses were performed with this RNA rapid extraction kit. Primers were designed with actin as the internal control gene using Primer 6.0 (Table S1). The cycling procedure included an initial denaturation step at 95 °C lasting 30 s, succeeded by 40 cycles of three-step amplification: 95°C for 5 s, 57 °C for 10 s, and 72 °C for 20 s, followed by a melt curve analysis. The gene’s relative expression was determined using the 2^−ΔΔCt^ method, and statistical variations were assessed with a significance threshold of *p* < 0.05.

### 2.4. Statistics and Analysis

The data were generated with Microsoft Excel 2021, assessed for significance through the DPS 7.05 software, and visualized with Origin 2021.

## 3. Result

### 3.1. Identification and Physicochemical Property Analysis of the Maize IPT Gene Family

**Figure 1:**
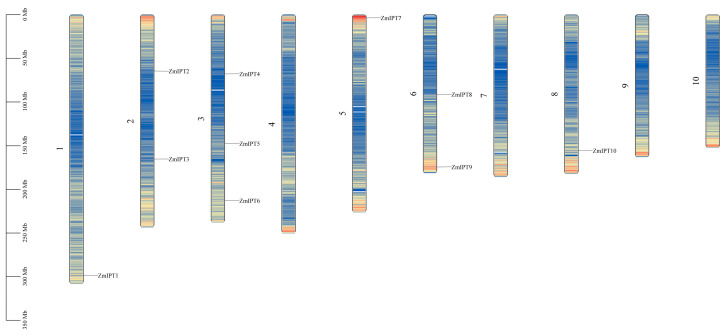
Distribution of the *ZmIPT* Gene Family on Chromosomes.

In the maize genome, the present researchers identified a total of ten members of the IPT family, labeled as *ZmIPT1* to *ZmIPT10*. The physical and chemical properties of the maize IPT proteins were analyzed, with results presented in Table 1. The average length of the maize IPT family amino acids was 368.1 amino acids (aa), and the average relative molecular mass was 39,778.19 Da. Among these, *ZmIPT5* exhibited the longest amino acid sequence and the highest relative molecular mass, while *ZmIPT4* displayed the shortest amino acid sequence and the lowest relative molecular mass. *ZmIPT1* and *ZmIPT2* are characterized by a positive charge, whereas the remaining proteins possess a negative charge. The average isoelectric point (pI) value is 8.13; with the exception of *ZmIPT2* and *ZmIPT4*, all other proteins have a pI greater than 7, suggesting a potential role in alkaline subcellular environments. TargetP-based subcellular localization predictions showed that most members of the IPT gene family (*IPT1*, *IPT3*, and *IPT5-IPT10*) are localized in the chloroplast, while only a few members (*IPT2* and *IPT4*) are localized in the cytoplasm, suggesting a considerable degree of functional divergence within the family at the subcellular level. Combined with the SignalP analysis, which indicated that all members have D-scores far below the threshold of 0.45, it can be inferred that these proteins lack typical N-terminal signal peptides and are unlikely to be secreted via the classical secretory pathway(see Table S2). Instead, they are likely to function within specific intracellular compartments-particularly in the chloroplast-where they may participate in cytokinin biosynthesis or regulation. In addition, chromosomal localization analysis revealed that the 10 *ZmIPT* genes are distributed across six different chromosomes, showing a relatively dispersed pattern within the maize genome (see Figure 1).

### 3.2. Phylogenetic Analysis of the ZmIPT Gene Family

**Figure 2:**
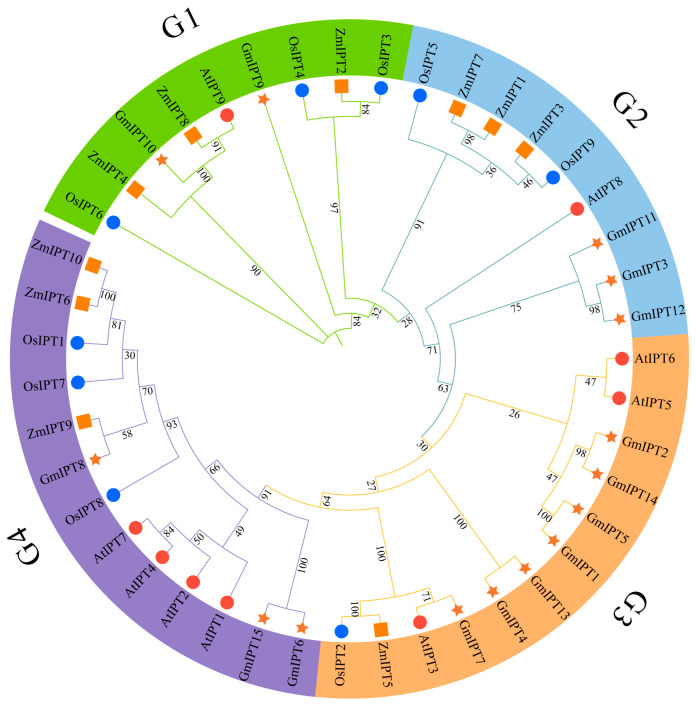
Phylogenetic Tree of the *IPT* Gene Family. The IPT members of Arabidopsis thaliana (At), rice (Os) and maize (Zm) are identified by red circles, blue circles, and yellow squares, respectively, and the numbers on the branches of the phylogenetic tree represent the self development values.

To further elucidate the evolutionary relationships within the maize *IPT* gene family, we selected 42 *IPT* protein sequences from maize (10), Arabidopsis (9), rice (9), and soybean (15) to construct a phylogenetic tree. The findings suggest that the 42 IPT proteins can be divided into four distinct subfamilies (G1–G4). Interestingly, the IPT genes found in maize are spread throughout all subfamilies, indicating substantial variations in the IPT protein family across different species. Additionally, the IPT families of maize and rice exhibit a closer homology relationship (Figure 2).

### 3.3. Analysis of Conserved Motifs in the ZmIPT Gene Family

**Figure 3:**
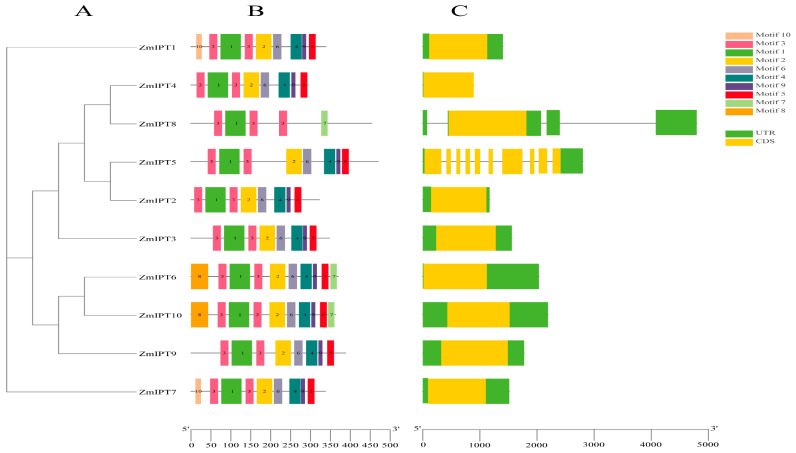
Evolutionary tree of the *ZmIPT* gene family. (**A**), Conserved Motifs (**B**), and Gene structure analysis (**C**).

We identified eight conserved motifs within the ZmIPT proteins. Notably, Motif1 and Motif3 are functional domains shared among all ten IPT protein sequences, suggesting that these two motifs may play a crucial role in the functions of family members. Additionally, *ZmIPT3* contains only four motifs, which is significantly fewer than those found in other genes, while *ZmIPT1* and *ZmIPT2* each contain the maximum of ten motifs. This disparity may reflect the structural and functional diversity among the different genes. Through clustering analysis, we observed that members with similar structures were grouped into the same subfamily (Figure 3A,B). All members of the *ZmIPT* gene family contain introns; however, only *ZmIPT5* possesses two UTR non-coding regions. This suggests that the genetic architecture of the maize *ZmIPT* gene family is comparatively straightforward, and genes belonging to the same subfamily might display analogous functions (Figure 3C).

### 3.4. Synteny Analysis of the ZmIPT Gene Family

**Figure 4:**
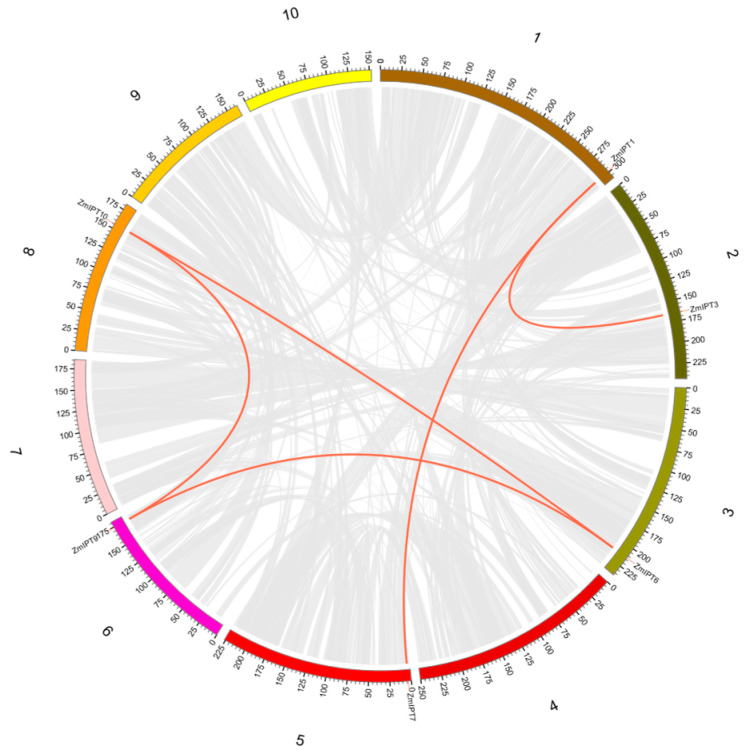
Synteny analysis of *ZmIPT* gene family.

TBtools was employed to perform a collinear analysis of the maize IPT gene family, with the findings presented in Figure 4. This analysis revealed a total of five gene pairs within the family: *ZmIPT1* with *ZmIPT3*, *ZmIPT1* with *ZmIPT7*, *ZmIPT6* paired with *ZmIPT9*, *ZmIPT6* associated with *ZmIPT10*, and *ZmIPT9* alongside *ZmIPT10*. Additionally, the genes exhibiting collinear relationships are categorized within the same subfamily, indicating their similar structural and functional characteristics. Further examination revealed that these collinear genes are part of the same subfamily, suggesting that they may have undergone gene duplication events during evolution. Consequently, these genes we found to hold significant importance for the functionality of the maize IPT family. Further analysis based on Ka/Ks ratios revealed that all five gene pairs have Ka/Ks values significantly lower than 1 (ranging from 0.05 to 0.20; detailed values provided in Supplementary Tables S3 and S4), indicating strong purifying selection during their evolutionary history. These results imply that these genes have conserved functions and likely play essential roles in the maize *IPT* gene family.

### 3.5. Analysis of Cis-Acting Elements in the Promoters of the ZmIPT Gene Family

**Figure 5:**
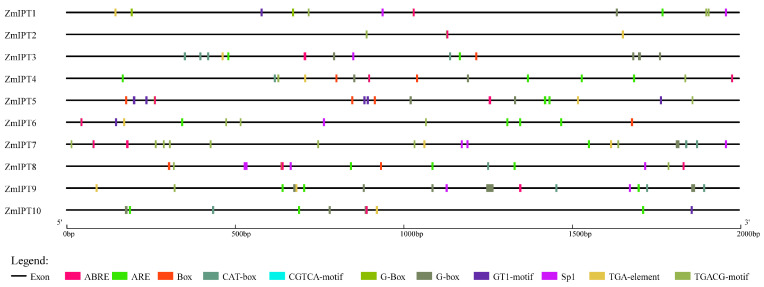
Distribution of Cis-Acting Elements in the *ZmIPT* gene family.

In this research, we performed an analysis of the cis-acting elements within the promoter region situated 2000 bp upstream of the maize *IPT* gene, as illustrated in Figure 5. The cis-acting elements identified consisted of elements responsive to light, elements associated with stress responses, those linked to plant growth and development, as well as elements related to plant hormone responses. Among the IPT genes analyzed, *ZmIPT2* exhibited the fewest cis-acting elements, while *ZmIPT7* contained the most. Notably, all *IPT* genes featured the abscisic acid response element ABRE, suggesting a significant role in regulating abscisic acid levels. With the exception of *ZmIPT8*, most IPT genes also possessed the auxin response element TGA, indicating their involvement in auxin regulation. Furthermore, all genes except for *ZmIPT2* contained anaerobic induction-related elements (ARE) and light-responsive elements (G-Box), demonstrating that the majority of these genes respond to environmental signals and light induction.

### 3.6. Expression Analysis of ZmIPT Genes in Different Tissues

**Figure 6:**
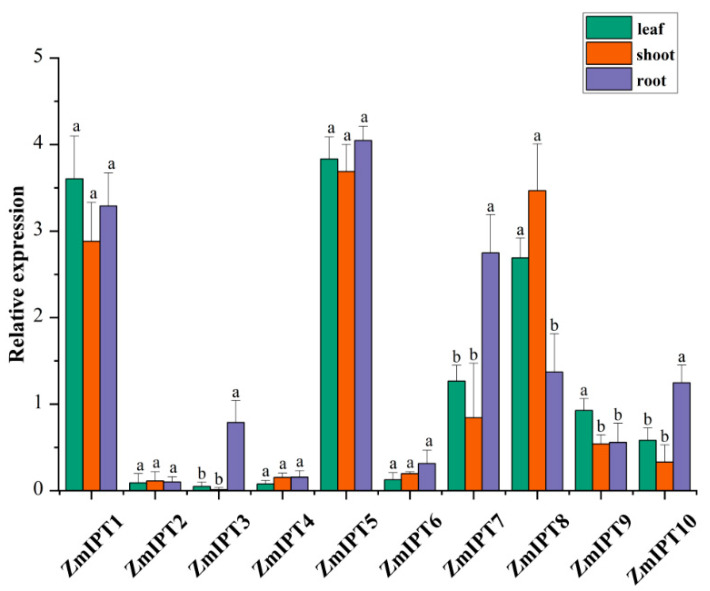
Tissue-Specific Expression Analysis of *ZmIPT* Gene family. The different letters in the figure represent significant differences with a *p*-value less than 0.05.

We analyzed the expression patterns of the maize *IPT* gene family across various tissues. The results indicate that the *ZmIPT5*, *ZmIPT7*, and *ZmIPT8* genes are highly expressed in the roots, particularly *ZmIPT5*, suggesting a significant role for these genes in maize root growth and hormone synthesis. In contrast, the expression levels of the *ZmIPT2* and *ZmIPT3* genes in stems and leaves were nearly undetectable. Additionally, the *ZmIPT4*, *ZmIPT6*, and *ZmIPT10* genes generally exhibited low expression levels. Overall, the observed differences in *IPT* gene expression across different tissues reflected the distinct functions of each gene in maize growth and development (Figure 6).

### 3.7. Expression Analysis of IPT Gene in Maize Under Abiotic Stress

**Figure 7:**
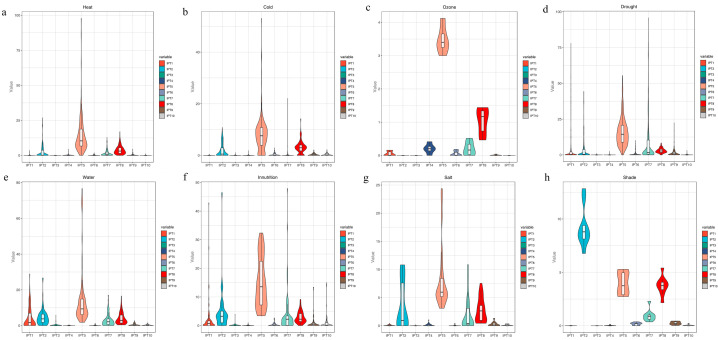
Abiotic stress expression analysis of *ZmIPT* gene family. (**a**) heat stress. (**b**) cold stress. (**c**) ozone stress. (**d**) drought stress. (**e**) water stress. (**f**) innutrition stress. (**g**) salt stress. (**h**) shade stress.

To investigate the response of *IPT* genes to abiotic stress in greater detail, we utilized a database to analyze the expression profiles associated with these genes. Our findings revealed distinct variations in the expression patterns of the *ZmIPT* genes. Notably, during exposure to both high and low temperature stress, the expression levels of *ZmIPT5* and *ZmIPT8* showed significant increases. In response to UV and drought stress, *ZmIPT5*, *ZmIPT7*, and *ZmIPT8* exhibited the highest expression levels. For nutritional stress, as well as saline-alkali and shade stress, *ZmIPT2*, *ZmIPT5*, *ZmIPT7*, and *ZmIPT8* displayed relatively high expression. Furthermore, *ZmIPT1* showed increased expression under flooding conditions. Importantly, *ZmIPT5* exhibited elevated expression levels across various abiotic stress conditions, highlighting its potential significance in mediating plant responses to these stressors (Figure 7).

**Figure 8:**
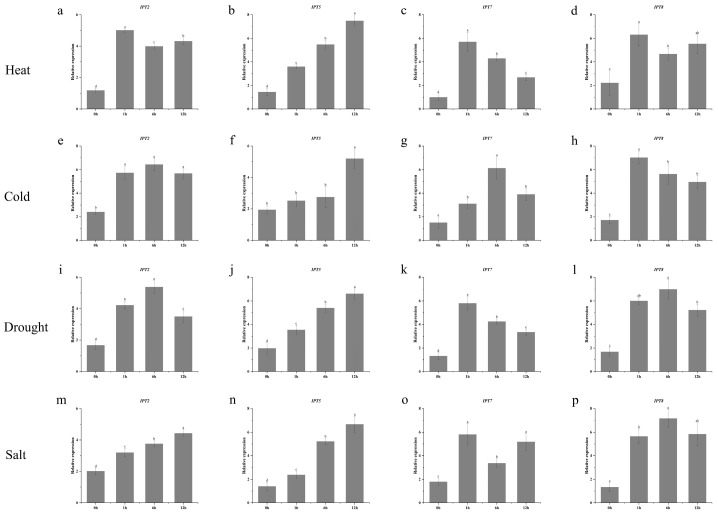
Expression analysis of *ZmIPT2*, *ZmIPT5*, *ZmIPT7*, and *ZmIPT8* under heat stress, cold stress, drought stress and salt stress at 0, 1, 6 and 12 h. (**a**–**d**) show the expression levels of *ZmIPT2*, *ZmIPT5*, *ZmIPT7*, and *ZmIPT8* under heat stress, while (**e**–**h**) depict the expression levels of these genes under cold stress. (**i**–**l**) present the expression levels of *ZmIPT2*, *ZmIPT5*, *ZmIPT7*, and *ZmIPT8* under drought stress, and (**m**–**p**) illustrate the expression levels of these genes under salinity stress.

To explore how *IPT* genes responded to abiotic stresses such as heat, cold, drought, and salt stress, we analyzed the spatiotemporal expression variations of these genes under different stress conditions using qRT-PCR. The results indicate that *ZmIPT2*, *ZmIPT7*, and *ZmIPT8* are rapidly upregulated during the early stages of stress (at 1 h or 6 h), suggesting that these genes may facilitate a swift plant response and adaptation to environmental changes by regulating cytokinin synthesis and signaling. In contrast, *ZmIPT5* exhibited relatively continuous high expression across all stress conditions, peaking at 24 h, which suggests its significant role in long-term adaptive responses. Specifically, the early response of *ZmIPT2* to both high and low temperatures, the sustained expression of *ZmIPT5* under various stresses, the initial activation of *ZmIPT7* under low and saline conditions, and the mid-term regulation of *ZmIPT8* during drought and salinity collectively indicate that these genes serve distinct biological functions in the plant’s responses to abiotic stress (Figure 8).

### 3.8. Prediction of Interacting Proteins of IPT5 in Maize

**Figure 9:**
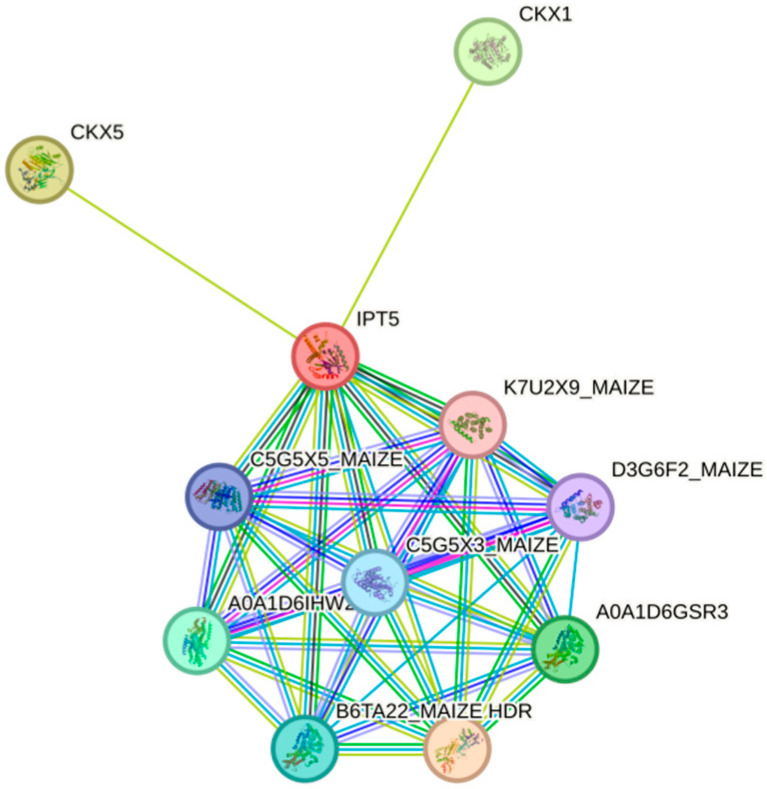
The interacting protein prediction of *ZmIPT5.* The colored nodes in the figure represent query proteins and the first shell of interactors, while the white nodes represent the second shell of interactors. Filled nodes indicate that a 3D structure is known or predicted. The light blue edges represent interactions from curated databases, purple-red edges represent experimentally determined interactions, green edges represent gene neighborhood, red edges represent gene fusions, blue edges represent gene co-occurrence, yellow edges represent text mining, black edges represent co-expression, and purple edges represent protein homology.

In our investigation of the interaction proteins of *ZmIPT5*, we identified that it is primarily associated with *CKX1*, *CKX5*, and proteins from the FPP/GGPP synthase family. *CKX1* and *CKX5* belong to the cytokinin oxidase family and are primarily responsible for the degradation of cytokinins (CKs). This association suggests that *ZmIPT5* may be involved in regulating cytokinin metabolism, playing a crucial role in maintaining the balance between cytokinin synthesis and degradation, which, in turn, influences plant growth and development-particularly in roots and other tissues where precise regulation of cytokinin levels is essential. Furthermore, the FPP/GGPP synthase family is primarily involved in the synthesis of farnesyl and geranylgeranyl pyrophosphates, which serve as precursors for plant growth hormones, secondary metabolites, and signaling molecules. The interaction with these enzymes indicates that ZmIPT5 may also have a regulatory function in hormone synthesis, contributing to plant developmental processes and responses to environmental stress (Figure 9).

## 4. Discussion

The *IPT* gene family has been recognized across various species [32,35,36,37], with many investigations revealing that *IPT* genes are essential for plant growth, development, and responses to environmental stresses [35,36]. However, the specific functions of *IPT* genes in maize remain unknown. This study systematically analyzed 10 members of the *IPT* family. Analysis of their physical and chemical properties reveals significant differences in the lengths of amino acid sequences and relative molecular masses among maize *IPT* family members, which may contribute to the functional diversity observed. Research indicates that most *IPT* genes are localized to the plastids, while a smaller number are found in the mitochondria [9]. In Arabidopsis, *AtIPT1*, *3*, *5*, and *8* are localized to plastids, *AtIPT4* is found in the cytoplasm, and *AtIPT7* is localized to the mitochondria [37]. In maize, eight *IPT* genes are located in the chloroplast, and two are situated in the cytoplasm, suggesting that maize IPT proteins function in both the chloroplast and cytoplasm. Most *IPT* genes exhibit isoelectric points greater than 7, indicating that they may function effectively in alkaline environments, which aligns with their distribution in the chloroplast and cytoplasm. As chloroplasts are crucial sites for plant hormone synthesis, this suggests that *IPT* genes play a fundamental role in regulating plant growth, photosynthesis, and stress responses. 

Through the construction of phylogenetic trees, we observed that maize *IPT* genes were distributed across various subfamilies, exhibiting significant homology. This indicates that *IPT* genes among different species share common evolutionary foundations in terms of structure [38]. Moreover, the proximity of maize to the rice *IPT* gene family may reflect their shared history of gene duplication and functional differentiation during evolution [39]. We identified eight conserved motifs, with Motif1 and Motif3 being present in all ten IPT protein sequences. The variation in the number of motifs among different genes—such as *ZmIPT1* and *ZmIPT2*, which contain the most motifs (ten), while *ZmIPT3* has only four—suggests functional differences among these genes, potentially correlating with their roles in plant growth and environmental responses. The variations in the characteristics of stimuli were also found to be interconnected. The primary factor behind the expansion of gene families in plants is the replication of gene fragments, which results in the development of unique functions following gene duplication [40]. Collinearity analysis revealed five pairs of collinear gene relationships, all within the same subfamily, indicating that these genes may have preserved similar functional properties throughout evolution [41]. Studies have demonstrated that *IPT* genes play a crucial role in regulating plant responses to adverse stresses. Identifying and characterizing cis-regulatory sequences is essential for processes like plant development and responses to environmental changes [42].

Research indicates that auxin can function as a signaling molecule, inducing the expression of stress-resistant genes when plants experience abiotic stress. For instance, during the reproductive phase of soybean, *GmIPT8* and *GmIPT10* are upregulated in response to drought stress [43]. The cis-acting element analysis conducted in this study has uncovered the potential roles of *IPT* genes in phytohormone signaling, stress responses, and growth and development processes. Importantly, the prevalent occurrence of ABRE and TGA elements suggests that the maize *IPT* gene family may play a role in the plant’s response to challenging conditions. Gene expression is modulated by transcription factors interacting with cis-acting elements found in promoter regions of genes [44]. To further analyze the expression patterns of *ZmIPT* genes, this study utilized public RNA-seq database data to conduct a preliminary analysis of 10 *ZmIPT* genes. The findings indicate that *AtIPT3*, *AtIPT5*, and *AtIPT7* genes in Arabidopsis exhibit stable and moderate expression across various tissues [36]. In rice, *OsIPT1* and *OsIPT2* are expressed at higher levels in roots, stem tips, and flowers. Our research indicated that the expression of *ZmIPT* genes in various tissues showed no considerable variations, with *ZmIPT5* being consistently expressed across all tissue types. This suggests that *ZmIPT5* could be involved at every stage of maize development.

In agricultural production, common abiotic stresses include extreme temperatures, salinity, drought, and flooding. Previous research has shown that salt stress can result in reduced cytokinin levels in Arabidopsis, thereby improving the plant’s ability to withstand salt stress [14]. We analyzed the expression of 10 *ZmIPT* genes in various tissues under conditions of extreme temperature, drought, and salt stress. Notably, *ZmIPT5* exhibited high expression under diverse abiotic stress conditions, suggesting its potential role in regulating plant responses to these stresses and highlighting its possible function as a key regulator in enhancing maize’s tolerance to adverse conditions. 

## 5. Conclusions

This study systematically identified 10 *IPT* family members (*ZmIPT1*–*ZmIPT10*) in the maize genome and comprehensively analyzed their gene structures, physicochemical properties, evolutionary relationships, expression patterns, and responses to abiotic stress. The findings not only clarified the structural evolution and regulatory characteristics of the *ZmIPT* gene family but also highlighted the functional roles of key genes—particularly *ZmIPT5*, which exhibited sustained high expression and broad involvement under stress conditions. These results significantly advance our understanding of cytokinin biosynthesis regulation in plants and provide valuable insights into the synergistic roles of phytohormones in abiotic stress tolerance. Moreover, the data and conclusions from this study offer important theoretical foundations and genetic resources for the molecular breeding of stress-tolerant maize varieties, contributing to the improvement of crop stability and resource-use efficiency under climate change conditions.

## Figures and Tables

**Table 1:** Information and Physicochemical Properties of the Maize *IPT* Gene Family Members.

Gene Name	Gene ID	Chromosomal Location	Protein Length	MW (kDa)	pI	Subcellular Localization Prediction
*ZmIPT1*	Zm00001eb062030	Chr1:298566258..298567661 (+)	338	36,488.4	9.04	chloroplast
*ZmIPT2*	Zm00001eb084710	Chr2:64584655..64585825 (+)	322	34,485.8	4.88	cytoplasm
*ZmIPT3*	Zm00001eb095250	Chr2:165161688..165163248 (−)	347	37,238	8.22	chloroplast
*ZmIPT4*	Zm00001eb131660	Chr3:67863267..67864159 (−)	294	31,953	5.92	cytoplasm
*ZmIPT5*	Zm00001eb139980	Chr3:147368450..147371548 (−)	470	52,240.5	7.34	chloroplast
*ZmIPT6*	Zm00001eb156350	Chr3:212809994..147371548 (−)	369	39,117.1	9.56	chloroplast
*ZmIPT7*	Zm00001eb212350	Chr5:3603911..3605426 (−)	337	36,571.7	9.97	chloroplast
*ZmIPT8*	Zm00001eb271810	Chr6:91352085..91357228 (−)	453	50,884.2	7.22	chloroplast
*ZmIPT9*	Zm00001eb294970	Chr6:174266460..174268235 (−)	388	40,656.8	8.98	chloroplast
*ZmIPT10*	Zm00001eb360550	Chr8:155530469..155532662 (−)	363	38,146.4	10.17	chloroplast

## Data Availability

The data are available upon request from the corresponding author.

## Supplementary Materials

The following supporting information can be downloaded at: https://www.mdpi.com/article/10.3390/genes16040415/s1, Table S1: Primer Design; Table S2: SignalP analysis and subcellular localization prediction of the IPT gene family; Table S3: Sequence of the IPT gene family; Table S4: KaKsanalysis of gene pairs.
